# Terbium to Quantum Dot FRET Bioconjugates for Clinical Diagnostics: Influence of Human Plasma on Optical and Assembly Properties

**DOI:** 10.3390/s111009667

**Published:** 2011-10-12

**Authors:** Frank Morgner, Stefan Stufler, Daniel Geißler, Igor L. Medintz, W. Russ Algar, Kimihiro Susumu, Michael H. Stewart, Juan B. Blanco-Canosa, Philip E. Dawson, Niko Hildebrandt

**Affiliations:** 1 NanoPolyPhotonik, Fraunhofer Institut für Angewandte Polymerforschung, D-14476 Potsdam, Germany; E-Mails: frank.morgner@iap.fraunhofer.de (F.M.); stefan.stufler@iap.fraunhofer.de (S.S.); 2 Physikalische Chemie, Universität Potsdam, D-14476 Potsdam, Germany; E-Mail: daniel.geissler@uni-potsdam.de; 3 Center for Bio/Molecular Science and Engineering, Code 6900, US Naval Research Laboratory, Washington, DC 20375, USA; E-Mails: igor.medintz@nrl.navy.mil (I.L.M.); russ.algar.ctr.ca@nrl.navy.mil (W.R.A.); 4 College of Science, George Mason University, 4400 University Dr., Fairfax, VA 22030, USA; 5 Optical Sciences Division, Code 5611, US Naval Research Laboratory, Washington, DC 20375, USA; E-Mails: susumu@ccs.nrl.navy.mil (K.S.); michael.stewart@nrl.navy.mil (M.H.S.); 6 Departments of Cell Biology and Chemistry, The Scripps Research Institute, La Jolla, CA 92037, USA; E-Mails: juanbautista.blanco@usc.es (J.B.B.-C.); dawson@scripps.edu (P.E.D.); 7 Institut d’Electronique Fondamentale, Université Paris-Sud 11, Orsay Cedex 91405, France

**Keywords:** FRET, quantum dots, terbium, luminescence lifetime, blood, plasma, clinical diagnostics, biotin, streptavidin, histidin, immunoassay

## Abstract

Förster resonance energy transfer (FRET) from luminescent terbium complexes (LTC) as donors to semiconductor quantum dots (QDs) as acceptors allows extraordinary large FRET efficiencies due to the long Förster distances afforded. Moreover, time-gated detection permits an efficient suppression of autofluorescent background leading to sub-picomolar detection limits even within multiplexed detection formats. These characteristics make FRET-systems with LTC and QDs excellent candidates for clinical diagnostics. So far, such proofs of principle for highly sensitive multiplexed biosensing have only been performed under optimized buffer conditions and interactions between real-life clinical media such as human serum or plasma and LTC-QD-FRET-systems have not yet been taken into account. Here we present an extensive spectroscopic analysis of absorption, excitation and emission spectra along with the luminescence decay times of both the single components as well as the assembled FRET-systems in TRIS-buffer, TRIS-buffer with 2% bovine serum albumin, and fresh human plasma. Moreover, we evaluated homogeneous LTC-QD FRET assays in QD conjugates assembled with either the well-known, specific biotin-streptavidin biological interaction or, alternatively, the metal-affinity coordination of histidine to zinc. In the case of conjugates assembled with biotin-streptavidin no significant interference with the optical and binding properties occurs whereas the histidine-zinc system appears to be affected by human plasma.

## 1. Introduction

Over the last decades, applications based on Förster Resonance Energy Transfer (FRET) have become very valuable tools for innumerable applications in the fields of medicine and biology [1–3]. Due to the *r*^−6^ distance dependence of FRET it is possible to gain access not only to small structural changes in biological processes such as protein folding but also to kinetic data of reactions (e.g., enzymatic activity) and binding events. FRET is used in both heterogeneous and homogeneous immunoassays. In most assay types, the FRET pairs consist of combinations of organic dyes and fluorescent proteins (FPs) [4–7]. Using the well-characterized organic dye family as FRET probes has the advantage of a small size, which alleviates some bioconjugation issues and guarantees a small impact on the biomolecule. They also exhibit long-term storage stability in a wide range of media and facile use [8]. FPs likewise are easy to attach to biomolecules and have little influence on biological systems [9]. Moreover, they can be expressed within the biological system of interest (e.g., for live cell imaging). Nevertheless, these advantages are accompanied by some drawbacks that must be kept in mind when performing FRET experiments, such as a relatively small Stokes-Shift, broad emission spectra and photobleaching. The luminescence lifetime of dyes and FPs is usually in the nanosecond time-range, so that signal and background emission can only be distinguished via spectral information, not via temporal characteristics. However, especially in biological media an accurate spectral discrimination is complex due to high background from autofluorescence [10,11]. In addition the Förster distances (donor-acceptor distance, where the FRET efficiency is 50%) rarely exceed 6 nm [12]. All these hindrances complicate clinical diagnostics, where reproducibility and accuracy of multiple parameter measurements are extremely important.

The FRET pair combination of luminescent terbium complexes (LTCs) as donors and semiconductor quantum dots (QDs) as acceptors overcomes the limitations mentioned above. High photostability, brightness and luminescence quantum yields, large Förster distances and an excellent temporal as well as spectral separation make this FRET-pair a powerful tool for multiplexed FRET measurements in a wide range of applications in research and diagnostics [13,14]. The long luminescence lifetime of the LTCs (in the milliseconds range) allows nearly background-free detection of the FRET-sensitized QD emission by time gated detection of fluorescence emission [15]. QDs were used as acceptors because of their suitability for multiplexed detection. The symmetric emission bands with a small FWHM (full width at half maximum) in combination with the tunable emission wavelengths largely facilitate the simultaneous determination of different analytes (so called multiplexing) compared to dyes or fluorescent proteins [16]. Although LTC-QD FRET pairs have proven to be very useful when performing biosensing in various buffer solutions, the very important aspect of measuring in real-life *in vitro* diagnostic media such as human plasma or serum has not yet been considered. However, the functionality of the FRET-pair must be preserved in these media in order to achieve the integration into many clinical diagnostics assays—a critical next step for utilizing these nanocrystalline materials.

Human blood is a complex cocktail of many different substances, which can be classified mainly into two components: cells (40–45% of volume) and plasma (55–60% of volume) [17]. As disease-specific biomarkers are contained in the plasma, this part of the blood is most interesting for clinical diagnostics from point-of-care to high-throughput-screening. In addition autofluorescence, scattering and absorption are strongly reduced compared to measurements in whole blood. Nevertheless these effects can still greatly bias measurements [18] and must therefore be carefully observed.

In this study we compare conjugation of LTCs to biocompatible QDs in buffer and plasma to evaluate the influence of plasma on photophysical properties and the sensing performance of several different LTC-QD-systems. For these purpose we chose two different conjugation systems, namely biotin (Biot) binding to streptavidin (sAv) and histidine (His) to zinc (Zn) metal affinity. LTC-conjugated sAv was binding to biotinylated QDs, whereas peptides functionalized with LTCs on one end and displaying a hexahistidine tag on the other end were assembled directly to the zinc-rich surface of DHLA-capped QDs.

## 2. Experimental Section and Methods

The LTC “Lumi4Tb” labeled to streptavidin (*ca.* 4.4 Lumi4Tb per sAv) was provided by Lumiphore Inc. (Richmond, CA, USA). For peptide labeling, *N*-hydroxysuccinimide-activated Lumi4-Tb (NHS-Lumi4-Tb) was provided by Lumiphore. The peptide utilized here consisted of the sequence H_2_N-G•SGAAAGLS•(His)_6_-amide and can be essentially subdivided into three modular components as indicated. The N-terminal amine extending from the G residue provides a unique site-specific handle for labeling with the NHS-Lumi4-Tb. The SGAAAGLS portion should form a short alpha-helix around the alanines which are then disrupted at each side by the glycines; this motif serves as a short intervening linker. Lastly, the C-terminal (His)_6_ provides for high-affinity assembly to the QDs while the amide blocks the terminal carboxyl. Overall, this peptide is meant to allow the Tb label to have close proximity to the QD for efficient FRET while not allowing direct contact with the QD surface. The peptide was labeled using the manufacturer’s suggested protocol and then purified, desalted, lyophilized and stored at −20 °C in a dessicator until used as previously described in detail [19].

Biotinylated QDots™ with emission maxima at 529 (Biot-QD529), 653 (Biot-QD653) and 712 nm (Biot-QD712), respectively, were purchased from Invitrogen Inc. (Carlsbad, CA, USA) with an average of six Bio/QD. These QDs are assumed to be surface functionalized with a proprietary amphiphilic polymer [20]. 530 nm emitting and 580 nm emitting CdSe/ZnS core/shell along with 615 nm emitting CdSe/CdS/CdZnS-alloy/ZnS multilayer or “onionskin” QDs were synthesized as described with some modifications [21]. These were then surface-functionalized and made water compatible with dihydrolipoic acid (DHLA) ligands as described. It is important to note, that the latter DHLA ligand imparts colloidal stability to the QD via its deprotonated terminal carboxyl, which has previously only allowed these materials to be used in basic media.

The solvents used were 10 mM TRIS-buffer (pH 7.4) without any further additives, 10 mM TRIS-buffer (pH 7.4) with 2% bovine serum albumin (BSA), and human plasma collected from fresh blood (pH ∼7.4). The latter were collected in accordance with all institutional regulations. The plasma was extracted from fresh blood before each measurement. For this purpose the blood was centrifuged for 30 minutes at 2,500 g directly after extraction and then the supernatant was used for measurements. Because of the strong plasma absorption (especially in the UV), it was diluted with water four times for absorption measurements. For all other measurements undiluted plasma was used. All experiments were performed at room temperature.

Absorption spectra were recorded in 1 cm quartz cells with a UV-VIS-spectrometer (Lambda35, PerkinElmer, USA). Luminescence (emission and excitation) spectra and lifetimes were recorded in 3 mm quartz cells with a fluorescence lifetime spectrometer (FLS920, Edinburgh Instruments, UK). For the lifetime measurements the samples were excited with a Xe flashlamp (50 Hz repetition rate at 340 nm) for LTCs and with a diode-laser (405.6 nm center wavelength, 2 MHz repetition rate, 5 mW maximum average power) for the QDs. FRET-measurements were performed on an immunoanalysis platereader (IOM Nanoscan LF500, Berthold Detection Systems, Germany) with two photomultiplier tube (PMT) detection channels using changeable bandpass filters (Semrock, USA). All samples were excited with a pulsed 337.1 nm nitrogen laser system (SpectraPhysics, USA) with 128 shots at 30 Hz repetition rate and a pulse energy of *ca*. 30 μJ (at the sample) for Biot-sAv conjugates and 100 μJ (at the sample) for His-Zn conjugates. For all samples the LTC and the QD emission were recorded simultaneously in the two PMT channels (LTC channel and QD channel). Decay time curves were recorded from 0 to 10 ms after the excitation pulse in 1,000 bins of 10 μs.

For all spectroscopic measurements the sample volume was 150 μL. All samples were mixed and incubated within the used media (QD-DHLA to LTC-pep assembly happened inside these media without pre-assembly). FRET-measurements were performed in 20 μL sample volume. Concentrations for absorption measurements were 1 μM for LTC-sAv, 10 μM for LTC-Pep, 50 nM for Biot-QDs and 100 nM for QD-DHLA. Luminescence measurements were performed with concentrations of 1 μM LTC-sAv or LTC-Pep and 100 nM for all QDs, respectively. Detection of FRET in the Biot-sAv conjugate assays was performed with QD concentrations of 0.08, 0.15, 0.2, 0.25, 0.3, 0.38, 0.5, 0.75, 1, 1.5 and 3 nM, respectively. The LTC-sAv concentration was kept constant at 3 nM. Detection of FRET in the His-Zn conjugate assays was performed with five times higher concentrations, *i.e.*, QD concentrations from 0.4 to 15 nM and a constant LTC-Pep concentration of 15 nM.

Luminescence decay times of LTCs, QDs and the FRET systems were calculated from the decay curves by multi-exponential fitting procedures (I = ∑(A_x_exp(−t/τ_x_)); LTCs: two variable exponentials; QDs: three variable exponentials; FRET-system acceptors: three variable exponentials; FRET-system donors: three variable exponentials and one fixed exponential corresponding to the decay time of the unquenched donor). Mathematical fits were performed with FAST™ software (Edinburgh Instruments).

## 3. Results and Discussion

For the evaluation of the influence of plasma on photophysical and binding properties, we first performed basic photophysical measurements with the donors and acceptors separately. Afterwards we conducted time-resolved and time-gated measurements of the different LTC-QD FRET pairs within Biot-sAv and His-Zn conjugate assays to show the influence of plasma on QD-based FRET for clinical assays. In order to state more precisely which of the components of plasma interact with the FRET partners, we performed comparative measurements in TRIS-buffer and TRIS-buffer containing BSA (at a mass fraction of two percent, which is comparable to the fraction of human serum albumin in blood plasma) and in human plasma.

### 3.1. Photophysical Characterization of FRET-Donors and -Acceptors

**Figure 1.**
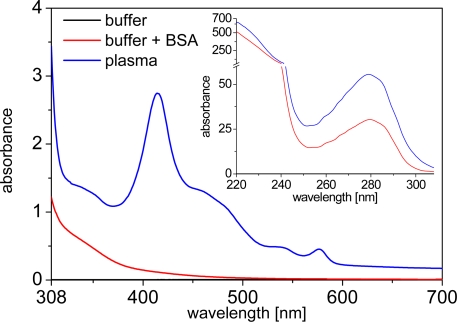
Absorbance spectrum (1 cm path length) of human plasma. The inset shows the absorbance below 308 nm, which was measured at 200-fold dilution and the absorbance values were multiplied by a factor of 200.

Human plasma is a medium, which shows extremely high absorbance in the UV (inset of Figure 1) due to the high concentration of proteins. Beyond ca. 310 nm the absorbance is much weaker, showing some typical peaks of hemoglobin absorption (Figure 1). Nevertheless, compared to fluorophores, which are usually highly diluted in clinical assays, the absorption of undiluted plasma is still large between 310 and 600 nm. This results in a simple attenuation of incoming excitation as well as outgoing emission light (at wavelengths shorter than 600 nm) that needs to pass through the plasma medium. Although this does not influence the photophysical or binding properties of the fluorophores, it needs to be taken into account for optical measurements.

**Figure 2.**
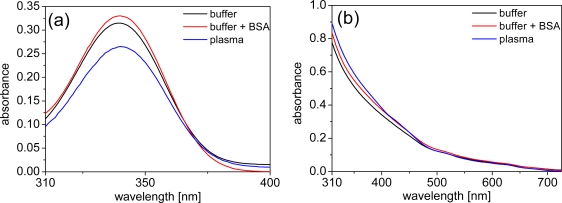
Representative absorbance spectra **(a)** LTC-Pep (c = 10 μM, corrected by absorbance of media, plasma diluted to ¼) **(b)** Biot-QD705 (c = 50 nM, corrected by absorbance of media, plasma diluted to ¼).

Due to the strong UV absorption, all of the following absorption spectra were measured beyond 310 nm. For both the LTCs and the QDs the absorption spectra within the different media (TRIS buffer, TRIS buffer + BSA and plasma) are unchanged and small differences in intensity arise due to the strong background of plasma or BSA absorption, which needed to be subtracted to show the pure absorption of LTCs and QDs. Using very highly concentrated solutions of LTCs or QDs would overcome this problem. However, the high costs of both these materials usually limit such types of measurements. Even with some intensity deviations due to the correction for plasma and BSA absorption, the most important aspect of unchanged shapes of the absorption spectra are still obvious (Figure 2).

**Figure 3.**
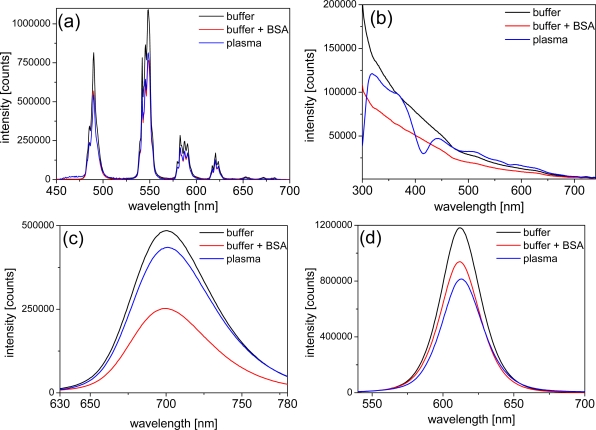
Emission and excitation spectra in buffer and plasma **(a)** LTC-Pep emission spectrum (c = 1 μM) **(b)** Biot-QD712 excitation spectra (λ_em_ = 705 nm, c = 50 nM) **(c)** Biot-QD712 emission spectra (λ_ex_ = 340 nm, c = 50 nM) **(d)** QD-DHLA615 emission spectra (λ_ex_ = 340 nm, c = 100 nM).

In order to obtain a deeper look into the possible influences of BSA and plasma on the photophysical properties of LTCs and QDs, we performed luminescence measurements of the different components. The negligible emission intensity of plasma allowed the use of undiluted plasma for all emission-based experiments. In order to avoid significant absorption of excitation and emission light by plasma we chose 3 mm cells. The emission intensity of LTC-Pep is almost equally quenched by BSA and plasma [*ca*. 30%–*cf*. Figure 3(a)] whereas the emission intensity of LTC-sAv is only weakly influenced (with a decrease of ca. 10% - data not shown). This effect can be explained by the fact that, in case of the LTC-sAv, the LTC is already attached to the sAv protein and thus protected from further influence of plasma proteins. The much smaller peptide complex of LTC-Pep is more influenced by the plasma proteins.

The QD emission spectra reveal an inconsistent picture of plasma and BSA influence. The strong absorption of undiluted plasma can still be seen very nicely within the different excitation spectra of the QDs [Figure 3(b)], where the absorption maximum of hemoglobin around 430 nm becomes visible as a minimum in the Biot-QD712 spectrum in plasma. For wavelengths beyond 500 nm the influence of plasma absorption on the excitation spectra is negligible. In the case of the Biot-QD712 luminescence quenching due to BSA is evident within the excitation and emission spectra [Figure 3(b,c)], whereas plasma only slightly decreases the emission intensity. The influence of BSA and plasma on the emission of the different Biot-QDs is not identical. For Biot-QD653 the influence of BSA and plasma on the emission intensities is almost negligible whereas the luminescence intensities of the Biot-QD529 decreases significantly from buffer to buffer+BSA to plasma (data not shown). We assume that static as well as dynamic quenching effects play different roles on these materials which are characterized by differently shapes (elongation of the QDs with increasing sizes [22]) and composition (QD529 and QD653: CdSe/ZnS; QD712: CdSeTe/ZnS). This was also confirmed by the fluorescence lifetime measurements (*vide infra*). The same inconsistent behavior can be found for the DHLA-capped QDs, where luminescence intensity quenching of plasma is very strong and BSA almost negligible for the smaller CdSe/ZnS QD-DHLA530 whereas the intensities are significantly quenched for both BSA and plasma for the larger CdSe/ZnS QD-DHLA580 (data not shown). The largest onionshell structured CdSe/CdS/CdZnS-alloy/ZnS QD-DHLA615 shows decreased emission intensity from buffer to buffer+BSA to plasma [Figure 3(d)].

**Figure 4.**
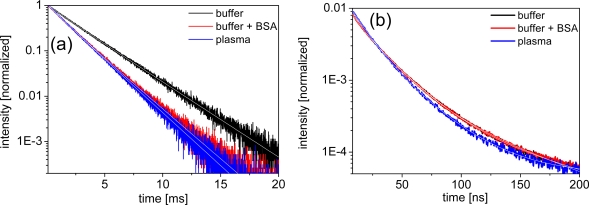
Luminescence decays of LTC donors and QD acceptors in buffer and plasma **(a)** LTC-Pep (λ_ex_ = 340 nm, λ_em_= 545 nm, accumulation time = 5 min, c = 1 μM) **(b)** QD-DHLA615 (λ_ex_ = 405.6 nm, λ_em_= 615 nm, accumulation time = 5 min, normalized to area under curve, c = 100nM).

Distinguishing the different quenching processes can be done by comparing the decrease in luminescence intensity to the decrease in luminescence lifetime. If the intensity ratio *I*_0_/*I* (unquenched to quenched intensity) equals the lifetime ratio τ_0_/τ, the quenching is purely dynamic. If *I*_0_/*I* > 1 and τ_0_/τ = 1, the quenching is purely static. If *I*_0_/*I* > τ_0_/τ > 1 the quenching is a combination of static and dynamic. Figure 4 shows typical luminescence decay curves of LTCs and QDs, respectively. The LTC decay curves show an almost mono-exponential in the millisecond lifetime range, whereas the QD decay curves are typically multi-exponential in the 1 to 100 nanoseconds regime. LTC curves were fit with a double-exponential function, whereas QD curves were fit with a triple-exponential. All lifetime data are gathered in Table 1.

Looking at the luminescence lifetime data of the LTC samples the trend is quite consistent with the intensity results. LTC-sAv shows a small decrease in lifetime by *ca.* 10% for both BSA and plasma and we thus assume a dynamic quenching process (*I*_0_/*I* = τ_0_/τ ≈ 10/9) due to interactions between LTC-sAv and the protein containing media. For LTC-Pep, the lifetime and intensity data indicate the same but stronger dynamic quenching process with *I*_0_/*I* = τ_0_/τ ≈ 10/7. As mentioned above this can be explained by the smaller size of the LTC-Pep, which is much more influenced by BSA and the plasma proteins than the LTC-sAv. It has been previously reported that BSA and human serum albumin (HSA) can form ternary complexes with LTCs, serving as stabilizers and additional antennas and thus increasing the luminescence intensity of such complexes [23,24]. Regarding our luminescence intensity and lifetime results, which point towards a dynamic quenching process, such behavior could not be found for the LTC complexes. However, in contrast to the enhancement experiments, where nM concentrations of BSA and HSA were used, our biological media contained μM concentrations of these components and at the same time nM concentrations of the FRET components.

For the different QDs the behavior of the lifetimes in the different media is again inconsistent. Only Bio-QD653 (paralleling the intensity data) is not significantly influenced by BSA and plasma within the experimental errors. For Biot-QD529 an increasing lifetime quenching can be observed from BSA to plasma, although the quenching is weaker, which points to a combination of static and dynamic quenching (*I*_0_/*I* > τ_0_/τ > 1). A similar trend can be found for QD-DHLA580 and QD-DHLA615 in plasma and QD-DHLA712 in BSA-buffer. QD-DHLA580 and QD-DHLA615 show the same lifetimes for TRIS-buffer with or without BSA, but the intensity is significantly quenched (stronger for QD-DHLA580). This behavior can possibly be explained by a static quenching (*I*_0_/*I* > 1 and τ_0_/τ = 1) due to attachment of BSA to the QD surface via electrostatic interaction. QD-DHLA530 shows an unexpected behavior of quenched intensity and increasing lifetime from buffer to BSA to plasma, which could result from a static attachment of proteins (denser protein layer for plasma compared to BSA) to the QD surface (intensity quenching) accompanied by a structural influence on the photophysical properties of the QD, leading to an increase of the longer time components within the multi-exponential QD decay. The same effect but with a much stronger increase of the long lifetime component accompanied by a strong decrease of the shortest one and a decent decrease of intensity can be found for the Biot-QD712 in plasma. Moreover, this is in complete contradiction to the behavior in BSA-containing buffer, where the average lifetime decreases similarly to the intensity.

Serum albumin is known to bind a lot of different substances through hydrophobic bonds and electrostatic interactions [25,26], and non-specific binding of plasma proteins, mainly of HSA, to QD surfaces has been shown before [27–29]. Nanoparticles in solution are stabilized by electrostatic and/or steric effects. The former increase with increasing surface charge, the latter increase with increasing particle size and functional surface groups, which can interact with the media [30]. In our spectroscopic study we could not find a clear trend of specific interactions of plasma components with the QDs and there was no clear difference between the interaction with the polymer coated large Biot-QDs and the DHLA surface-capped rather small QD-DHLAs. We conclude that our measurements fit to the somehow general picture of semiconductor quantum dots within aqueous or biological media: not every QD is alike [31].

### 3.2. FRET-Assays

Immunoassays are very important for clinical diagnostics. Homogenous assays allow fast, inexpensive and highly sensitive detection, because they do not require any time-consuming washing and separation steps [32]. FRET can be used to detect biomolecular recognition processes and is one of the most sensitive detection methods for homogeneous immunoassays [33]. Besides high sensitivity and specificity, two challenges are of paramount importance for homogeneous FRET assays: the suppression of autofluorescence from the biological media and the possibility of measuring several parameters simultaneously (multiplexing). Both of these aspects can be efficiently fulfilled by LTCs, which offer long luminescence lifetimes and thus the possibility of time-gating and sufficient suppression of background, and QDs, which can be used in different colors as FRET acceptors with LTCs and thus offer the possibility of multiplexing [16].

#### 3.2.1. Specific Binding Assays with Biotin and Streptavidin

**Figure 5.**
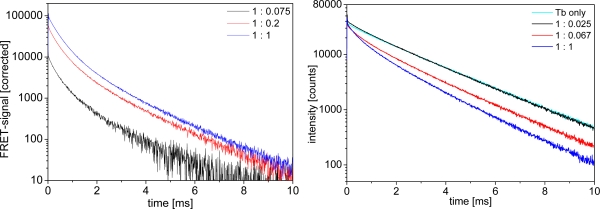
Decay curves of LTC-sAv and Biot-QD653 in plasma, LTC concentration c = 3 nM, increasing QD concentration (1:0.025 = 0.075 nM, 1:0.067 = 0.2 nM and 1:1 = 3 nM) **(left)** QD acceptor channel (background corrected) **(right)** LTC donor channel.

Biot-sAv binding is a widely used model system for specific biological binding because of the high binding affinity (dissociation constant of the biotin-streptavidin binding is about 10^−13^ M [34]). Adding Biot-QDs to LTC-sAv leads to a quenching of the LTC luminescence and the appearance of new, shorter, time components, distinguishable by the change of the donor decay curves from an almost mono-exponential behavior (with max. 10% of a shorter time component) for LTC alone to a multi-exponential shape. These shorter lifetime components are the FRET-quenched decay times. The related FRET-sensitized decay times for the QD-acceptors are visible in the acceptor luminescence curves, which increase in intensity due to FRET-sensitization. Figure 5 shows representative LTC-to-QD FRET luminescence decay curves in the different media at different concentrations. All decay curves show the same trend of FRET-sensitized QD emission appearing in the acceptor channel (Figure 5 left) and FRET-quenched LTC emission in the donor channel (Figure 5 right).

**Figure 6.**
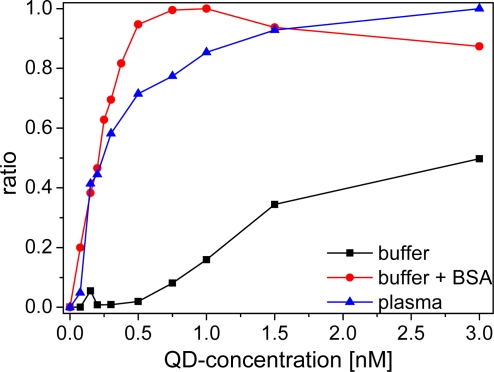
Ratio of sensitized acceptor emission and donor emission I_A_/I_D_ for LTC-sAv (3 nM) and Biot-QD653 (signal intensity integrated over time window 0.01–10 ms).

An important aspect for the sensitivity and reproducibility of time-resolved homogeneous FRET assays is the calculation of the ratio of time-gated emission intensities of the donor and the acceptor [16]. A so-called ratiometric measurement calibration curve displays the intensity ratio as a function of the analyte concentration, or in our case the QD acceptor concentration at a fixed LTC donor concentration (Figure 6). The calibration curve displays a steep rise of the ratio for BSA-buffer and plasma measurements after addition of small amounts of Biot-QD. The curve reaches a plateau at a sAv to Biot concentration ratio of *ca.* 6:1 in BSA-buffer, in good agreement with the average amount of six Biot molecules on the QD surface. In plasma this saturation effect is less abrupt and occurs later (around unity). For pure TRIS buffer measurements, no saturation is observable within the measured concentration range. The difference in ratios is caused by a different increase of sensitized acceptor luminescence with increasing QD concentrations. In plasma and BSA-buffer the polystyrene walls of the microwell plates are saturated with proteins and non-specific binding of Biot-QD or LTC-sAv is strongly suppressed [35,36] and all the FRET partners are free in solution. In protein-free buffer this non-specific binding is not suppressed and the FRET signal is much lower for small LTC-sAv-Biot-QD concentrations. We assign the difference in the calibration curves of BSA and plasma to the many different components within the plasma, which can non-specifically bind to the QD surface. This stronger non-specific binding compared to BSA causes a less efficient binding of LTC-sAv to Biot-QD and thus saturation at higher concentrations.

The limits of detection (LODs) of all LTC-sAv-Biot-QD systems were calculated by using the concentration value (within the linearly rising part of the calibration curves) of the blank ratio (no Biot-QDs) plus three times the standard deviation of 30 blank measurements (*cf*. Table 2). All Biot-QD systems show sub-nanomolar LODs in protein containing media. The pure buffer values are significantly higher due to the non-specific binding problems discussed above. The LODs are in agreement with the weaker luminescence quenching effects for Biot-QD653 and 712 in plasma compared to Biot-QD529 (*vide supra*), for which also the LOD is significantly higher for plasma measurements. The systems of LTC-sAv and Biot-QD653 and Biot-QD705 are very well suited for clinical assays because their detection limits are in the low picomolar range.

#### 3.2.2. Metal Affinity Coordination Assays with Zinc and Polyhistidine

In contrast to the specific sAv-Biot interactions, we utilize metal affinity coordination between His-Zn as an alternate QD bioconjugation method. A growing number of groups have demonstrated that this interaction can be exploited for assembling proteins, peptides and even DNA to QDs functionalized with a number of different types of surfaces or ligands [20,38]. Polyhistidine binds with high affinity to certain metal ions, including especially nickel, copper, cobalt, manganese, iron and especially zinc. The binding is a result of the sharing of electron density between histidine’s imidazole nitrogen with the electron-deficient orbitals of transition metals. The binding between polyhistidine and the zinc on QD surfaces is known to be extremely stable with a dissociation constant of ∼1 × 10^−9^ M [39]. Using the Zn^2+^-rich surface of QDs and hexahistidine-tagged peptide assembly we have successfully applied this mechanism to create a number of different FRET and charge transfer based biosensors that were capable of monitoring DNA hybridization, enzymatic activity and pH changes [2,38,40].

**Figure 7.**
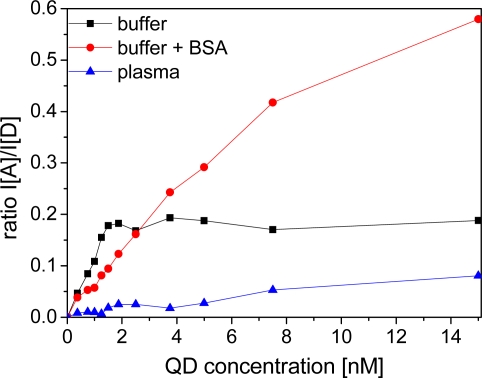
Ratio of sensitized acceptor emission and donor emission I_A_/I_D_ for LTC-Pep (15 nM) and QD615-DHLA (signal intensity integrated over time window 0.01–10 ms).

In TRIS buffer and BSA-containing buffer the addition of small amounts of QD-DHLA to a solution of LTC-Pep leads to an immediate FRET-sensitization of the QD-acceptor emission demonstrating an efficient binding of the histidine-tagged LTC-peptides to the QD surface. Although experiments have shown that up to 50 ± 10 peptides can bind to the surface of the QD [41], we found a saturation at ca. 10 peptides/QD within our ratiometric calibration curves, which might be caused by the low concentrations applied in our assays, being close to the dissociation constant (*ca*. 1 nM) of the hexahistidine-peptide-QD binding system [39]. Thus, our saturation value is rather a natural binding equilibrium than a maximum occupation of binding sites. The higher dissociation constant compared to Biot-sAv could lead to a lower fraction of quenched donors, because most of the donors and acceptors are unbound in solution. Nevertheless, the still relatively high ratio of donors per acceptor after addition of small amounts of QDs leads to a significant FRET-signal increase (Figure 7). Analogous to the Biot-sAv assays, FRET leads to sensitized acceptor emission and quenched donor emission and the time-gated intensity ratios are recorded.

Due to the much lower dissociation constant for Biot-sAv binding, the LODs of the QD-DHLA-LTC-Pep systems are significantly larger compared to the Biot-sAv systems. As the QD-DHLA-LTC-Pep system does not include any proteins, non-specific binding to the well walls is not a problem and thus the addition of BSA does not have a strong influence on the LODs, which even slightly increase with addition of BSA. Surprisingly, and in contrary to the photophysical properties, which showed almost no difference between buffer with or without BSA, QD-DHLA530 revealed a very bad assay performance in pure TRIS-buffer but relatively low LODs within BSA-containing buffer; reinforcing the hypothesis that individual QD responses can change drastically due to subtle surface perturbations.

For all QD-DHLA-LTC-Pep systems in plasma no trace of FRET could be observed even at equivalent ratios of donors and acceptors neither for acceptor nor for donor emission. The small increase of ratio over QD concentration (Figure 7) is caused by the strong fluorescence from directly excited QDs. This signal can still be measured after hundreds of microseconds. As the influence of plasma on the photophysical properties of the single components is not at all sufficiently strong to explain such a behavior, we assign this lack of FRET to a very inefficient binding of the His_6_-tag of the donor to the ZnS-shell caused by a plasma component. Human serum albumin can be excluded because measurements in BSA-containing buffer show very obvious FRET signals. Therefore other compounds of the plasma must prevent the binding. Most likely transferrin and the histidine-rich glycoprotein, which can be found in physiological concentrations of 60 μM and 2.5 μM, respectively, are responsible for this. These proteins are known to bind to Zn^2+^ with a dissociation constant of ∼10^−6^ M [42,43] and can compete successfully with the polyhistidine-tag of the peptide-labeled LTC, which is present in the assay at ca. three orders of magnitude lower concentrations. The prevention of the His-Zn binding in plasma needs to be taken into account, when this binding system is applied for clinical diagnostics.

## 4. Conclusions

FRET from LTCs as donors to QDs as acceptors in blood plasma has, for the first time, been described in this study. Two different conjugation systems were used to simulate two prominent types of QD assembly. A photophysical characterization of all donors and acceptors was performed in TRIS buffer, BSA-containing TRIS buffer, and fresh human plasma. The LTCs were mainly influenced by a dynamic quenching process due to interactions with BSA or the plasma proteins. The QDs gave a much more inconsistent picture of interactions with BSA or plasma with a combination of static and dynamic quenching mixed with some structural stabilization effects. However, all these different influences could not be clearly assigned to the different QD properties, thus leading us to the conclusion that even though there are similarities in size, shape or material composition, no QD is alike. Specific binding assays used conjugates assembled with biotinylated QDs and streptavidin-labeled LTCs, whereas conjugates based on His-Zn binding were realized with DHLA-capped QDs and peptide-labeled LTCs. Besides some unexpected effects for the smaller QDs, the principal finding is that for direct use in plasma and similar types of environments, QD assemblies should be initially based on biotin-avidin interactions or other alternative covalent chemistries [44].

The results for DHLA-capped QD assemblies in plasma were not entirely unexpected for a number of interrelated reasons. Given that experiments were performed at pH 7.4, where such QDs can become colloidally unstable in conjunction with using sample concentrations that are at the His-Zn dissociation point did not initially allow for anywhere near optimal conditions. Moreover, the DHLA can be charged which would also serve to attract oppositely charged species and contribute to fouling of the surface. Most critically, our experiments (focusing on a simple direct assay) utilized a format where the nanocomplexes were assembled stepwise in the plasma itself. Namely, the media was aliquoted, then the QDs were added followed by the LTC-pep in the last step and left to incubate for 1 hour. It is most probable that this configuration allowed a number of proteins and other biological molecules and ions to interact with and potentially block the QD surface. Moreover, plasma is known to have high concentrations of ions (Mg, Fe, *etc.*) that can and will also interact with and block the polyhistidine’s coordination and binding capacity.

These results, however, do not broadly discount assemblies based on His-Zn from such media, they rather suggest that either this assembly order or the DHLA ligand itself may be unsuitable for such media. More pertinently, we know of no assay formats that have successfully utilized DHLA QDs directly in plasma or blood. In contrast, initial QD assembly using His-Zn interactions have already been shown to be viable in the highly reducing intracellular environment and similar QD conjugates have been shown to remain assembled within the highly acidic cellular endolysosomal system of live cells for several days; this used QDs displaying either amphiphilic polymers or poly(ethylene glycol) (PEG) surfaces, respectively [45,46]. PEG ligands are broadly used in this field to functionalize QDs and make them water soluble. They provide two inherent benefits: (1) solubility is mediated by the uncharged ethylene oxide repeats allowing broad pH stability and (2) they are inherently antifouling. A further benefit in the current context is that their large size sterically prevents proteins, but not peptides, and other large molecules from accessing the QDs Zn surface [21]. Cumulatively, this should prevent charged species and proteins from fouling the QD surface and competing to remove assembled His-appended peptides. Another possibility in this direction is the use of QDs functionalized with compact zwitterionic ligands; these are also known for their broad pH stability and antifouling characteristics [21]. Future experiments will focus on improving CdSe/ZnS-polyhistidine viability in plasma by testing of QDs displaying these improved surfaces, more judicious control over relative QD concentrations in assays, iterative characterization of pre-assembly versus direct assembly in plasma along with extending the number of histidine repeat residues which should improve binding to the QD surface to an even higher affinity [39].

## Figures and Tables

**Table 1.** LTCs: Luminescence lifetimes (τ_x_) and intensity fractions (IF) of the single components and intensity averaged lifetime (<τ>). QDs: Luminescence lifetimes (τ_x_), intensity averaged lifetime (<τ>) and Förster distances (*R*_0_) with the LTC-conjugates.

	**Media**	**τ_1_ (μs)**	**IF (%)**	**τ_2_ (μs)**	**IF (%)**	**<τ> (μs)**
**LTC-Pep**	**Buffer**	1,373	6	2,620	94	2,545
	**Buffer + BSA**	1,179	16	1,983	84	1,852
	**plasma**	1,118	9	1,863	91	1,794
**LTC-Strep**	**Buffer**	941	9	2,606	91	2,455
	**Buffer + BSA**	933	10	2,390	90	2,249
	**plasma**	899	10	2,370	90	2,227
	**Media**	**τ_1_ (ns)**	**τ_2_ (ns)**	**τ_3_ (ns)**	**<τ> (ns)**	***R*_0_ (nm)**
**Biot-QD529**	**Buffer**	9	51	137	69	5.4
	**Buffer + BSA**	9	36	61	61	
	**plasma**	9	30	58	53	
**Biot-QD653**	**Buffer**	17	66	205	85	10.7
	**Buffer + BSA**	17	66	203	85	
	**plasma**	22	63	303	75	
**Biot-QD712**	**Buffer**	13	77	205	93	11.1
	**Buffer + BSA**	10	70	192	65	
	**plasma**	18	98	266	161	
**QD-DHLA530**	**Buffer**	5	20	100	33	7
	**Buffer + BSA**	6	23	112	43	
	**plasma**	5	22	117	47	
**QD-DHLA580**	**Buffer**	7	22	100	25	7.8
	**Buffer + BSA**	6	21	98	24	
	**plasma**	7	20	96	10	
**QD-DHLA615**	**Buffer**	9	26	106	36	7.9
	**Buffer + BSA**	11	27	121	19	
	**plasma**	11	24	140	31	

**Table 2.** Limits of detection (LODs ± 20% deviation) for the different LTC-QD FRET pairs for the specific (Biot-sAv) and metal affinity coordination (His-Zn) assays in the different biological media.

	
	**LOD in nM**		
**LTC-sAv**	**buffer**	**buffer + BSA**	**plasma**
Biot-QD529	0.27	0.0064	0.30
Biot-QD653	0.51	0.0047	0.018
Biot-QD712	0.13	0.0017	0.0026

**LTC-Pep**			

QD-DHLA530	5.2	0.63	-
QD-DHLA580	2.6	2.9	-
QD-DHLA615	0.11	0.28	-
